# The efficacy and safety of electro-acupuncture for alleviating chemotherapy-induced peripheral neuropathy in patients with coloreactal cancer: study protocol for a single-blinded, randomized sham-controlled trial

**DOI:** 10.1186/s13063-019-3972-5

**Published:** 2020-01-09

**Authors:** Kaiyin Chan, Louisa Lui, Kaling Yu, Kwongwai Lau, Manchi Lai, Waiwai Lau, Bacon Ng, Linda L. D. Zhong, Zhao-Xiang Bian

**Affiliations:** 1 Yan Chai Hospital cum Hong Kong Baptist University Chinese Medicine Clinic cum Training and Research Centre, Ha Kwai Chung, Hong Kong SAR; 2 Oncology Department of Princess Margaret Hospital, Kwai Chung, Hong Kong SAR; 3 Chinese Medicine Department, Hong Kong Hospital Authority, Kowloon, Hong Kong SAR; 4 Hong Kong Chinese Medicine Clinical Study Centre, Hong Kong Baptist University, Jockey Club School of Chinese Building, 7 Baptist Road, Kowloon Tong, Hong Kong SAR; 5 School of Chinese Medicine, Hong Kong Baptist University, Jockey Club School of Chinese Building, 7 Baptist Road, Kowloon Tong, Hong Kong SAR

**Keywords:** Acupuncture, Chemotherapy-induced peripheral neuropathy, Oxaliplatin, Colorectal cancer

## Abstract

**Background:**

Colorectal cancer is the most common cancer in Hong Kong. Oxaliplatin-based chemotherapy is a major first-line conventional therapy for advanced and metastatic colorectal cancer. However, oxaliplatin causes chemotherapy-induced peripheral neuropathy (CIPN). Acupuncture has long been used to alleviate limb numbness in Chinese medicine. This study aims to examine the efficacy and safety of acupuncture for alleviating CIPN in patients with colorectal cancer in Hong Kong.

**Methods/design:**

This is a single-blinded, randomized, sham-controlled efficacy trial. Eighty-four eligible patients, who are Hong Kong Chinese, aged ≥ 18 years, diagnosed with colorectal cancer and undergoing oxaliplatin-based chemotherapy, will be randomized in a ratio of 1:1 to the electro-acupuncture group or the sham-controlled group. During a 12-week treatment period, patients in the electro-acupuncture group will undergo electro-acupuncture once a week from the first cycle of chemotherapy, while patients in the control group will receive sham acupuncture, and the patients in both groups will be followed up for 12 weeks. The primary outcome measure is the Functional Assessment of Cancer Therapy/Gynecologic Oncology Group-Neurotoxicity (FACT/GOC-Ntx) questionnaire. The secondary outcome measures include numerical rating scale (NRS) for numbness/pain, vibration and light touch sense test, the European Organization for Research and Treatment of Cancer Quality of Life Questionnaire-C30 (EORTC QLQ-C30) and Constitution of Chinese Medicine Questionnaire (CCMQ).

**Discussion:**

The study will compare electro-acupuncture with sham acupuncture to explore the feasibility for electro-acupuncture in improving symptoms caused by chemotherapy-induced peripheral neuropathy.

**Trial registration:**

Clinicaltrials.gov, NCT03582423. Registered on 11 July 2018.

## Background

Colorectal cancer is the most common cancer in Hong Kong. In 2016, there were 5437 new cases and 2089 patients died due to colorectal cancer. The crude death rate from colorectal cancer in Hong Kong was reported as 28.5 per 100,000 [1]. Current conventional therapies include surgery, chemotherapy, radiotherapy, targeted therapy, and immunotherapy. Surgery is the most common treatment for all stages of colorectal cancer and patients with advanced stage cancer are usually given chemotherapy or radiotherapy to kill any possible remaining cancer cells. Oxaliplatin-based chemotherapy, regimens such as FOLFOX with or without (±) bevacizumab, CapeOX ± bevacizumab or FOLFOX ± panitumumab (KRAS wild type gene only), is one of the major first-line treatments for advanced or metastatic colorectal cancer [2]. However, 90% of patients with oxaliplatin develop acute chemotherapy-induced peripheral neuropathy (CIPN) [3], which occurs in 68.3% patients within 2.5 ± 1.1 (mean ± standard deviation) chemotherapy cycles [4]. Symptoms include paresthesia and dysesthesia of the hands, feet and perioral area, induced by cold stimuli [5]. About 30–50% of patients develop chronic CIPN after repetition of chemotherapy cycles [3] and its symptoms include paresthesia, numbness, sensory ataxia, functional deficits and pain. The mechanism of CIPN is complicated. Cytokine and chemokine binding are one of its possibilities. Chemotherapeutic agents enhance release of cytokines (e.g. TNF-α, IL-1β) and binding of chemokines to their receptors located on neurons and glial cells*,* which contributes to increased sensation of pain [6]. Although symptoms induced by CINP are not vegetative, coasting effect is observed, meaning that the symptoms continue to develop and progress for several months after the therapy, and a maximum duration of 8 years was documented [5]. Decrease in the levels of pro-inflammatory cytokines TNF-α, IL-1α, IL-1β induced by electro-acupuncture is thought to be related to its analgesic effect and thus its possibility to treat CINP.

According to traditional Chinese medicine theory, CIPN is similar to *Xue Bi*(血痹), which means pain and numbness in the extremities [6]. Herbal medicine and acupuncture are common treatments for *Xue Bi*. Systematic reviews of the CIPN found that the effectiveness of current treatments (including natural products and complementary therapies) are still unknown and only vitamin E is shown to possibly help alleviate CIPN [7, 8]. The current studies of acupuncture in CIPN vary in the use of different cancer and anti-cancer drugs such as taxanes for breast cancer [9], bortezomib and thalidomide for multiple myeloma [10], and a mix of neurotoxic anticancer drugs [11]. However, studies of acupuncture for oxaliplatin-induced peripheral neuropathy are rare and there has been no randomized controlled trial [12]. Therefore, we designed this study for a single-blinded, randomized controlled clinical trial to explore the efficacy and safety of acupuncture in relieving CIPN.

### Objective

The aim of this study is to assess the efficacy and safety of electro-acupuncture compared to sham acupuncture in alleviating CINP in patients with colorectal cancer.

## Methods/design

### Study design

**Fig. 1:**
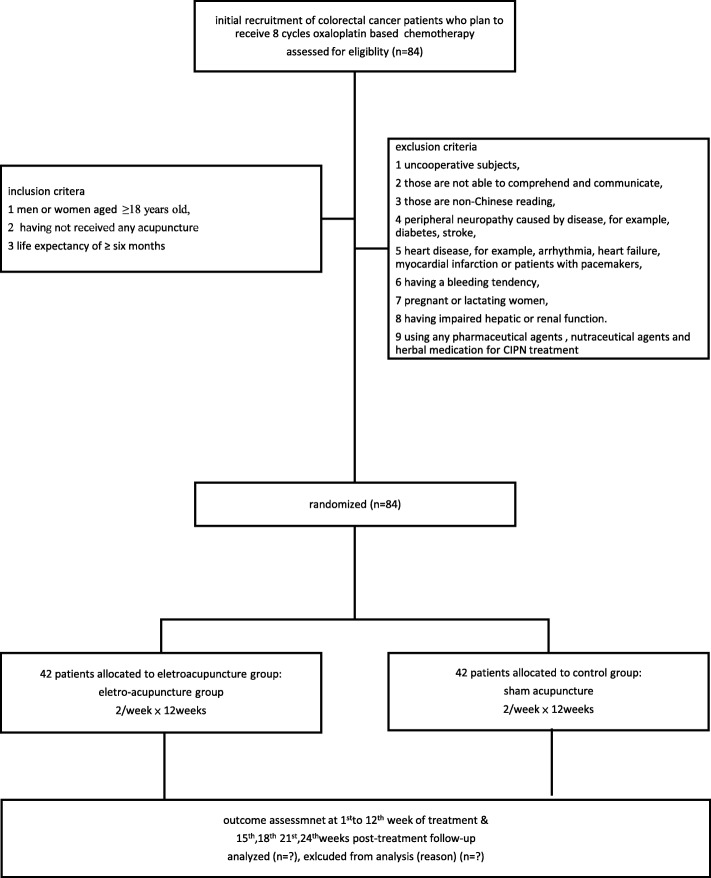
Participant flow diagram. CIPN, chemotherapy-induced peripheral neuropathy

This is a single-blinded, randomized, sham-controlled efficacy trial on electro-acupuncture for alleviating symptoms of chemotherapy-induced peripheral neuropathy in patients with colorectal cancer. A total of 84 candidates will be recruited in this study. They will be assigned randomly into either the electro-acupuncture or sham-control group. Both groups will be given a total of 12 sessions of interventions, with 1 session per week. They will then be followed up regularly for up to 12 weeks after the completion of the intervention. The Functional Assessment of Cancer Therapy/Gynecology Oncology Group-Neurotoxicity (FACT/GOG-Ntx) questionnaire (Additional file 1) will be used as the primary outcome measure. The questionnaire will be completed before the start of the study to provide a baseline score. The score will be obtained on a weekly basis during the intervention and every 3 weeks during the follow-up period, constituting a total of 24 weeks (Fig. 1). This study protocol has been approved by Hong Kong Baptist University Ethics Committee on the Use of Human Subjects for Teaching and Research (approval number HASC/17–18/C05) and Hospital Authority Kowloon West Custer Research Ethics Committee(KWC REC) (approval number KW/FR-18-041(121–01)) and registered in ClinicalTrials.gov (NCT03582423). The Standards for reporting interventions in controlled trials of acupuncture (STRICTA) 2010 [13] checklist for items is given in Table 1.

**Table 1:** Checklists for items in STRICTA 2010

Item	Detail
• Acupuncture rationale	1a) Style of acupuncture According to systematic reviews and clinical experiences of our principal investigator and co-investigators. Manual and electro-acupuncture based on traditional Chinese medicine theory
	1b) Reason for treatment provided, based on historical context, literature sources, and /or consensus methods, with references where appropriate
	1c) Extent to which treatment was varied: Standard treatment is used. No variation of treatment among patients
• Details of needling	2a) Number of needle insertions per subject per session (mean and range where relevant): 28 needles
	2b) Names (or location if no standard name) of points used (uni/bilateral) he gu 合谷(LI4), nei guan 內關(PC6), qu chi 曲池(LI12), ba xie 八邪(EX-UE9), zu san li 足三里(ST36), san yin jiao 三陰交(SP6), tai chung 太衝(LV3), ba feng 八風(EX-LE10)
	2c) Depth of insertions, based on a specified unit of measurement or on a particular tissue level: 10–25 mm
	2d) Response sought (e.g. de qi or muscle twitch response): De qi
	2e) Needle stimulation (e.g. manual, electrical): Manual and electrical - continuous waves at 2 Hz
	2f) Needle retention time: 25 min
	2 g) Needle type (diameter, length and manufacturer or material): Disposable acupuncture needles (verum acupuncture needles Hwato 0.25 × 0.25 mm matching the Streiterger sham needles)
• Treatment regimen	3a) Number of treatment sessions: 12 sessions
	3b) Frequency and duration of treatment sessions: Acupuncture will start after the day of 1st chemotherapy cycle 1/week for 12 consecutive weeks even if the chemotherapy is stopped
• Other components of treatment	4a) Details of other interventions administered to acupuncture group (e.g. moxibustion, cupping, herbs, exercise, lifestyle advice): No other intervention
	4b) Setting and context of treatment, including instructions to practitioners, and information and explanations to patients: Chinese Medicine Clinic cum Training and Research Centre Patients will be informed about acupuncture treatment in the study as follows: “in this study, acupoints for CINP will be used based on related reports and clinical experience of our investigators.”
• Practitioner background	5) Description of participating acupuncturists (qualification or professional affiliation, years in acupuncture practice, other relevant experience): Hong Kong registered Chinese medicine practitioners with at least 3 years of clinical experience and scholarship training in Chinese medicine oncology of Hong Kong Hospital Authority, who have undergone training and are able to provide identical acupuncture treatment in accordance with a predefined protocol
• Control or comparator interventions	6a) Rationale for the control or comparator in the context of the research question, with source that justify this choice: To assess the efficacy and safety of electro-acupuncture compared to sham acupuncture
	6b) Precise description of the control or comparator. If sham acupuncture or any other type of acupuncture-like control is used, provide details as for items 1 to 3 above Style of acupuncture Sham acupuncture Number of needle insertions per subjects per session: 28 Sham needles at the same acupoints as the treatment group Depth of insertion: Needles are only adhered to the skin Needle retention time: 25 min Needle type Streitberger’s non-invasive acupuncture needles (gauge 8 × 1.2″/ 0.30 × 0.30 mm) Number of treatment sessions: 12 sessions Frequency and duration of treatment sessions: 1/week for 12 consecutive weeks

This checklist, which should be read in conjunction with the explanations of the Standards for reporting interventions in controlled trials of acupuncture (STRICTA) items, is designed to replace item 5 in the Consolidated standards of reporting trials (CONSORT) 2010 when reporting an acupuncture trial

The study design also follows the Standard Protocol Items: Recommendations for Interventional Trials (SPIRIT) Checklist (Additional file 7).

### Participants and setting

Patients with colorectal cancer who are to receive oxaliplatin-based chemotherapy and do not have any pre-existing peripheral neuropathy will be recruited. The study is conducted in the Yan Chai Hospital cum Hong Kong Baptist University Chinese Medicine Clinic cum Training and Research Centre (Ha Kwai Chung).

### Inclusion criteria

Patients will be eligible for the study if they:

1. Are aged ≥ 18 years old
2. Are newly diagnosed with stage II–IV colorectal cancer
3. Are to receive eight cycles of adjuvant oxaliplatin-based chemotherapy
4. Having not received any acupuncture in the previous 3 months, and have life expectancy ≥ 6 months

### Exclusion criteria

Patients will be excluded from the study if they:

1. Are not able to comprehend and communicate.
2. Fail to cooperate with the researcher.
3. Are not able to read Chinese.
4. Having prior peripheral neuropathy caused by other diseases including diabetes mellitus, stroke, cardiovascular diseases such as arrhythmia, heart failure, and myocardial infarction, and patients with cardiac pacemakers.
5. Having a tendency to bleed easily
6. Are pregnant or breast-feeding.
7. Having impaired hepatic or renal function.
8. Are using any pharmaceutical agents e.g. vitamin B6 and vitamin E, or herbal medication for CIPN treatment. Any of these medications that are prescribed by physicians or Chinese medicine practitioners during the study will be recorded. Investigators will determine whether they need to be withdrawn from the study.

Candidates will be considered as dropouts from the study if they:

1. Withdraw his/her informed consent.
2. Are lost to follow up.
3. Develop a serious adverse event (SAE) or there are other safety/efficacy issues in which suspension will be considered beneficial as suggested by the investigators.
4. Become pregnant.
5. Stop taking the randomized treatment for any reason:
  The date and reason for withdrawal should be noted in the case report form (CRF). All subjects who withdraw from the study should, if all possible, continue to regularly attend follow up according to the schedule of measurements and be seen for a final evaluation (termination visit). Prematurely discontinued subjects will not be replaced.

### Recruitment

Subjects will be recruited through either of the following sources: (1) advertisements in newspapers; patients who are interested can contact research staff by phone; or (2) patients will be identified through clinic lists at the Princess Margaret Hospital (PMH) oncology outpatient department and Yan Chai Hospital cum Hong Kong Baptist University Chinese Medicine Clinic cum Training and Research Centre (Ha Kwai Chung). Potential participants will be approached initially by the relevant clinical team and then screened by research staff. Informed consent will be obtained from eligible patients (Additional file 6).

### Interventions

#### Electro-acupuncture treatment

The acupuncture intervention will be conducted for one session per week over 12 consecutive weeks. The most frequently used acupuncture points in CIPN are ba feng (EX-LE10), ba xie (EX-UE9), tai chung (LV3), he gu (LI4) [14]. Among them, the traditional effects of the points in hands and feet are regulating Qi and blood circulation and treating localized problems. With the clinical experience of our principal investigator and co-investigators, eight acupoints are chosen: he gu (LI4), nei guan (PC6), qu chi (LI12), ba xie (EX-UE9), zu san li (ST36), san yin jiao (SP6), tai chung (LV3), and ba feng (EX-LE10). The use of ba xie (EX-UE9) and ba feng (EX-LE10) will be optional if skin lesions on the hands and feet occur due to the use of capecitabine (Xeloda). The details of acupoints and their functions are listed in Table 2. The acupuncture treatment will be conducted by a registered Chinese medicine practitioner with more than 5 years of Chinese medicine college education plus at least 5 years of clinical experience. Disposable acupuncture needles (verum acupuncture needles Hwato 0.25 × 25 mm matching the Streiterger sham needles) will be inserted at a depth of 10–25 mm at the points. We will deliver electrical stimulation with continuous waves at 2 Hz, at an intensity of each patient’s minimum sensation of stimulation through the electrical acupuncture stimulation instrument (KWD808I multi-purpose health device, Ying Di, Chang Zhou, China) to the points. The needles will be retained in position for 25 min.

**Table 2:** Acupoints of electro-acupuncture and their location

Acupoint	Location
he gu (LI4)	In the middle of the 2nd metacarpal bone on the radial side
nei guan (PC6)	2 cun*above the wrist crease between the tendons of palmaris longus and flexor carpi radialis
qu chi (LI11)	At the lateral end of the transverse cubital crease midway between LU5 and the lateral epicondyle of the humerus
ba xie (EX-UE9)	On the dorsum of the hand, at the webs between each finger, at the junction of red and white skin
tai chung (LV3)	On the dorsum of the foot in a depression distal to the junctions of the 1st and 2nd metatarsal bones
san yin jiao (SP6)	3 cun directly above the tip of the medial malleolus on the posterior border of the tibia
zu san li (ST36)	3 cun below ST35, one finger width lateral from the anterior border of the tibia
ba feng(EX-LE10)	On the dorsum of the foot between the web and metatarsophalangeal joint (4 points on each foot)

*Cun is a standard measurement used to locate acupoints. It varies by patient and is equal to the width of the distal inter-phalangeal joint pf the thumb

#### Sham acupuncture

For subjects assigned to the control group, Streitberger’s non-invasive acupuncture needles (gauge 8 × 1.2″/0.30 × 30 mm) will be applied as a sham control at the same acupoints, which are used in the acupuncture intervention group, as the same stimulation modality, except that the needles are only adhered to the skin by a small plastic ring instead of being inserted [15, 16] and the stimulation will be a “pseudo stimulation”, which will be given by connecting the needle to an incorrect output socket of the electrical acupuncture stimulation instrument. The credibility and validity of this system has been well-demonstrated [17, 18].

### Outcome measures

The primary outcome is the validated Functional Assessment of Cancer Therapy/Gynecology Oncology Group/Neurotoxicity (FACT/GOC-Ntx) questionnaire (Additional file 1) [19, 20]. The questionnaire includes 11 questions covering sensory neuropathy, motor neuropathy, hearing neuropathy, and dysfunction associated with neuropathy. It results in a cumulative score ranging from 0 to 44, with the lower scores reflecting worse neuropathy symptoms. The secondary outcomes include (1) the numerical rating scale (NRS) (Additional file 2) for numbness/pain score in hands and feet [20], in which patients will be asked to rate their average neuropathy symptoms within 1 week, on a scale of 0–10 scale (0 = no symptoms, 10 = worst possible symptoms); those scoring < 4 out of 10 in the NRS will be considered as having mild CIPN while those scoring ≥ 4 will be considered as having moderate to severe CIPN [21]; (2) the validated European Organization for Research and Treatment of Cancer (EORCTC) quality of life questionnaire QLQ-C30 (Additional file 3) [22], which is a 30-item questionnaire assessing five functional scales (physical, role, cognitive, emotional, and social), three symptoms scales (fatigue, pain, nausea and vomiting), and other symptoms and problems experienced by patients with cancer (dyspnea, appetite loss, insomnia, constipation, diarrhea, and financial difficulties); (3) the validated Constitution of Chinese Medicine Questionnaire (CCMQ) (Additional file 4) [23, 24], which has 60 items measuring the nine body constitution types: gentleness, Qi-deficiency, Yang-deficiency, Yin-deficiency, phlegm-wetness, wetness-heat, blood-stasis, Qi-depression, and special diathesis; (4) the vibration sense test, by which the participant is assessed using the graduated Rydel-Seiffer tuning fork (U.S. Neurologicals, Poulsbo, WA, USA), with printed directions for use and its normative data [25]; readings will be averaged and recorded as the vibration value; and (5) the light touch test (Additional file 5), by which the participant is assessed with a fiber comprising a bundle of standard 10-g monofilaments contained within the Neuropen (Owen Mumford, Woodstock, UK). During testing, the fiber will be applied perpendicular to the plantar surface of the great toe and the palmar surface of the index finger until the fiber begins to bend, when it will be held in place for 1 s and then removed. This will be repeated three times and the patient will be asked to report if they can feel the fiber when it is applied [25].

The use of the aforementioned assessment questionnaires (FACT/GOC-Ntx, QLQ-C30, and CCMQ) will require authorization from the authors. In addition to the application of these measurements at baseline (0 weeks), the FACT/GOC-Ntx 11 items subscale, NRS, vibration sense test and light touch test will be assessed every week, the EORTC QLQ-C30 will be assessed every 3 weeks, and the CCMQ will be assessed at the end of treatment (12th week). The post-trial access by phone or face-to-face interview will be performed at the 15th, 18th, 21^st^, and 24th week. The schedule of evaluations is presented in Table 3. Adverse events will be noted throughout the study, based on the participants’ reports and routine laboratory tests (complete blood counts, renal and liver functions) before every chemotherapy cycle. Subjects will undergo routine laboratory tests of complete blood counts and renal and liver function before every chemotherapy cycle, at the hospitals where they received chemotherapy. Laboratory reports will be collected and thus any adverse events will be reported and noticed. All clinical adverse events will be recorded according to intensity (mild, moderate, or severe), duration, outcome, and relationship to the study.

**Table 3:** Schedule for outcome measurement

Period	B	T												F			
Chemo cycle		1st			2nd			3rd			4th			5th	6th	7th	8th
week		1	2	3	4	5	6	7	8	9	10	11	12	15	18	21	24
Informed consent	✓																
FACT/GOC-Ntx	✓	✓	✓	✓	✓	✓	✓	✓	✓	✓	✓	✓	✓	✓	✓	✓	✓
NRS	✓	✓	✓	✓	✓	✓	✓	✓	✓	✓	✓	✓	✓	✓	✓	✓	✓
EORTC QLQ-C30	✓			✓			✓			✓			✓	✓	✓	✓	✓
CCMQ	✓												✓				✓
Vibration sense test	✓	✓	✓	✓	✓	✓	✓	✓	✓	✓	✓	✓	✓				
Light touch test	✓	✓	✓	✓	✓	✓	✓	✓	✓	✓	✓	✓	✓				
Safety assessment	✓	✓	✓	✓	✓	✓	✓	✓	✓	✓	✓	✓	✓				

*B* baseline, *T* treatment phase, *F* follow-up phase, *FACT/GOC-Ntx* Functional Assessment of Cancer Therapy/Gynecologic Oncology Group-Neurotoxicity, *NRS* numerical rating scale, *EORTC QLQ-C30* European Organization for Research and Treatment of Cancer Quality of Life Questionnaire-C30, *CCMQ* Constitution of Chinese Medicine Questionnaire

### Randomization assignment

All subjects will be randomly assigned to either the intervention or the sham-controlled group. Subjects in the intervention group will receive electro-acupuncture treatment, whereas the subjects in the sham-controlled group will receive the sham treatment. A simple, non-sequential list of random numbers will be generated by a computer program prior to randomization. Randomization will be carried out by the research unit of the School of Chinese Medicine, Hong Kong Baptist University. The randomization numbers will be contained in sealed, opaque, and sequentially numbered envelopes and those envelopes will be stored in a locker and the key kept by the principal investigator (PI). The envelopes that correspond to the group allocation will be provided to the acupuncturist by the PI after completion of the subject recruitment procedure. Both the clinical assessor and subjects are thus blinded to the group allocation. If there are medical concerns leading to an inevitable review of treatment assignment, the PI will be the responsible person for approval. The date, time, and reason for treatment assignment disclosure should be noted in the case report form (CRF). Upon disclosure of their treatment assignment, the subject will be retained in the study. However, the number of cases in which randomization assignment is disclosed will be reported and, if necessary, sensitivity analyses may be performed after omitting these participants.

### Sample size calculation

The sample size calculation is based on change in the primary outcome. In this study, the difference in FACT/GOC-Ntx score between the intervention group and the sham-control group accounts for the calculation. As shown in a recent systematic review [12], there was only one study showing that acupuncture has significantly reduced the FACT GOC-Ntx score (mean difference = 5.40, SD = 3.91, 95% CI = 0.54–10.26) [26]. In this study, we assume the effect size between electro-acupuncture and sham acupuncture is 0.3. A minimum sample size of 62 should therefore be provided in order to achieve a significance level of α = 0.05 with power (1-β) of 80% (the number of measurements is 3 and correlation among repeat measures is 0.5) with calculation by Gpower 3.1 (*F* tests, analysis of variance (ANOVA), repeated measures, between factors). After taking a 25% dropout rate into consideration, the number of subjects needed for this study is 84.

### Data processing and analysis

Descriptive data including the rates of recruitment, dropout, and number of interventions missed will be analyzed and reported as count and percentage. All missing values, efficacy, and safety analyses will be based on the modified intention-to-treat (ITT) principle. The Statistical Package for the Social Sciences (SPSS) for Windows version 21.0 will be used for the statistical analysis. The statistical significance is defined as a two-sided *P* value <0.05. Baseline characteristics will be reported as mean and SD for continuous variables and count and percentage for categorical variables. The differences in both the normally distributed and non-normally distributed variables between the intervention and the sham-control groups will be assessed using Student’s *t* test and the Mann-Whitney *U* test, respectively. The chi-squared test or Fisher’s exact test will be used for calculating the categorical variables instead. Analysis of covariance (ANCOVA) will be used to compare the two groups every week, with treatment group as a factor in the model and baseline as the covariate. The difference between the baseline and endpoint score will be tested using repeated measures ANOVA. Any deviation from this original statistical plan will be described and justified in the final report.

### Data management and confidentiality

Personal data will be handled by the investigators. Hard-copy data will be stored in a secure locker and electronic data will be stored in a specified computer with encryption. The key for the locker and the password for the database will be kept by the investigators only. Personal data will be kept for 3 years after the study. Hard copies will be discarded as confidential waste, while the soft copy would be deleted and unrecoverable after completion of the study.

### Data monitoring and trial steering committee

Quarterly monitoring meeting will be held between the PI, co-investigators, the Hospital Authority Chinese Medicine Department (HACMD), and the School of Chinese Medicine, HKBU. The HACMD would monitor the Chinese Medicine Centre for Training and Research to ensure the quality of the trial and keep track of the study progress and compliance to the required standard and agreement.

## Discussion

This single-blinded, randomized, controlled, efficacy clinical trial aims at evaluating the efficacy and safety of acupuncture in chemotherapy-induced peripheral neuropathy (CIPN) in patients with colorectal cancer in Hong Kong. Although acupuncture is widely used in Hong Kong, studies on the alleviation of CIPN by acupuncture are lacking. Therefore, this study also aims to serve as an initiative for a non-pharmacological approach in improving such condition.

If the outcome from this study shows improvement in CIPN, it is hoped that further large-scale studies, perhaps with the combination of Chinese herbal medicine and in different types of cancer, could be conducted. Secondary outcome data, including the quality of life and Chinese medicine body constitution types, could also possibly inform further investigations in Chinese medicine theory.

The selection of acupoints is considered the major limitation, according to Chinese medicine theory. In this study, the selection of acupoints is standardized and utilized to every subject. Standardization of the acupoints provides easy and straightforward treatment implementation but is at the same time a potential limitation. Selection is not based on syndrome differentiation in Chinese medicine theory, which in other words, may result in over-simplification in treatment choice.

In conclusion, this single-blinded, randomized, controlled clinical trial will be conducted to evaluate the efficacy and safety of acupuncture on CIPN in Hong Kong. This study will explore the possibility for CMPs to utilize acupuncture for CIPN and will also provide a platform to offer research training opportunities for junior CMPs.

## Trial status

This study protocol version number is 4, dated 26 February 2019. The participants have been recruited for the present study since October 2018. Thirty-three patients are under treatment and recruitment will be completed in December 2019.

## Supplementary information

**Additional file 1:** FACT-GOG-NTX Questionnaire.

**Additional file 2:** Numerical Rating Scale.

**Additional file 3:** QLQ-C30 Questionnaire.

**Additional file 4:** CCMQ Questionnaire.

**Additional file 5:** Light Touch Test and Vibration Test.

**Additional file 6:** Information Sheet and Consent Form.

**Additional file 7:** SPIRIT 2013 Checklist.

## Data Availability

The datasets used and analyzed during the current study are available from the corresponding author on reasonable request.

## Abbreviations

ANCOVA: Analysis of covariance
ANOVA: Analysis of variance
CCMQ: Constitution of Chinese Medicine Questionnaire
CIPN: Chemotherapy-induced peripheral neuropathy
CMPs: Chinese medicine practitioners
EORTC QLQ-C30: European Organization for Research and Treatment of Cancer Quality of Life Questionnaire-C30
FACT/GOC-Ntx: Functional Assessment of Cancer Therapy/Gynecologic Oncology Group-Neurotoxicity
ITT: Intention to treat
NRS: Numerical rating scale
STRICTA: Standards for reporting interventions in controlled trials of acupuncture
