# Supplementary material for: The efficacy and safety of electro-acupuncture for alleviating chemotherapy-induced peripheral neuropathy in patients with coloreactal cancer: study protocol for a single-blinded, randomized sham-controlled trial
Source: Trials. 2020 Jan 9;21:58. doi: 10.1186/s13063-019-3972-5 (PMC6953283; doi:10.1186/s13063-019-3972-5)
Supplement: Supplementary file 1 — Additional file 1: FACT-GOG-NTX Questionnaire. [file 13063_2019_3972_MOESM1_ESM.pdf]

FACT/GOG-Ntx (Version 4)

以下是那些跟您有同樣疾病的人所認為重要的一些陳述。**請在每一行圈出或標出一個數字，以表達適用於您過去 7 天的回答。**

	生理健全狀況	一點也不	有一點	有些	相當	非常
GP1	我精神不好	0	1	2	3	4
GP2	我有反胃噁心的情形	0	1	2	3	4
GP3	因為我的身體狀況，我有困難達到家人的需求	0	1	2	3	4
GP4	我有疼痛	0	1	2	3	4
GP5	我對治療的副作用感到困擾	0	1	2	3	4
GP6	我覺得身體不適	0	1	2	3	4
GP7	我因病被迫要臥床休息	0	1	2	3	4

	社交/家庭健全狀況	一點也不	有一點	有些	相當	非常
GS1	我覺得與我的朋友親近	0	1	2	3	4
GS2	我從我家人獲得情緒上的支持	0	1	2	3	4
GS3	我從我朋友獲得支持	0	1	2	3	4
GS4	我家人已接受我的疾病	0	1	2	3	4
GS5	我滿意家人之間對我疾病的溝通方式	0	1	2	3	4
GS6	我覺得與我的伴侶 (或我主要支持者) 親近	0	1	2	3	4
Q1	*不管你近期的性生活的程度，請回答下面的問題。如果你不願回答，請在這裡註明 ☐ 然後跳到下一部分。*					
GS7	我對我的性生活感到滿意	0	1	2	3	4

Copyright 1987, 1997

FACT/GOG-Ntx (Version 4)

請在每一行圈出或標出一個數字，以表達適用於您過去 7 天的回答。

	情緒健全狀況	一點也不	有一點	有些	相當	非常
GE1	我感到悲傷	0	1	2	3	4
GE2	我滿意自己處理疾病的方式	0	1	2	3	4
GE3	我逐漸失去對抗我的疾病的希望	0	1	2	3	4
GE4	我覺得緊張	0	1	2	3	4
GE5	我擔心死亡	0	1	2	3	4
GE6	我擔心我的狀況會惡化	0	1	2	3	4

	功能健全狀況	一點也不	有一點	有些	相當	非常
GF1	我能夠工作 (包括在家的工作)	0	1	2	3	4
GF2	我滿意我的工作（包括在家的工作）	0	1	2	3	4
GF3	我能夠享受生活	0	1	2	3	4
GF4	我已接受我的疾病	0	1	2	3	4
GF5	我睡得好	0	1	2	3	4
GF6	我依然享受我以前常做的有趣的事	0	1	2	3	4
GF7	我滿足我現在的生活品質	0	1	2	3	4

Copyright 1987, 1997

FACT/GOG-Ntx (Version 4)

請在每一行圈出或標出一個數字，以表達適用於您過去 7 天的回答。

	附加關注事項	一點也不	有一點	有些	相當	非常
NTX 1	我的手有麻木或刺痛的感覺	0	1	2	3	4
NTX 2	我的腳有麻木或刺痛的感覺	0	1	2	3	4
NTX 3	我覺得雙手不舒服	0	1	2	3	4
NTX 4	我覺得雙腳不舒服	0	1	2	3	4
NTX 5	我有關節疼痛或者肌肉抽筋的症狀	0	1	2	3	4
HI12	我感覺渾身無力	0	1	2	3	4
NTX 6	我的聽力有問題	0	1	2	3	4
NTX 7	我會聽到耳朵裡有鈴聲或嗡嗡聲	0	1	2	3	4
NTX 8	我扣鈕扣有困難	0	1	2	3	4
NTX 9	我難以感覺手中小物體的形狀	0	1	2	3	4
An6	我走路有困難	0	1	2	3	4

Copyright 1987, 1997
