# Supplementary material for: The efficacy and safety of electro-acupuncture for alleviating chemotherapy-induced peripheral neuropathy in patients with coloreactal cancer: study protocol for a single-blinded, randomized sham-controlled trial
Source: Trials. 2020 Jan 9;21:58. doi: 10.1186/s13063-019-3972-5 (PMC6953283; doi:10.1186/s13063-019-3972-5)
Supplement: Supplementary file 2 — Additional file 2: Numerical Rating Scale. [file 13063_2019_3972_MOESM2_ESM.pdf]

Numerical rating scale (NRS)

請在每一行圈出一個數字，以表達適用於您過去7天的回答。

1. 我的手有麻木或刺痛的感覺

0	1	2	3	4	5	6	7	8	9	10
沒有麻木刺痛										你能想像麻木刺痛的最差程度

2. 我的腳有麻木或刺痛的感覺

0	1	2	3	4	5	6	7	8	9	10
沒有麻木刺痛										你能想像麻木刺痛的最差程度
