# Supplementary material for: The efficacy and safety of electro-acupuncture for alleviating chemotherapy-induced peripheral neuropathy in patients with coloreactal cancer: study protocol for a single-blinded, randomized sham-controlled trial
Source: Trials. 2020 Jan 9;21:58. doi: 10.1186/s13063-019-3972-5 (PMC6953283; doi:10.1186/s13063-019-3972-5)
Supplement: Supplementary file 3 — Additional file 3: QLQ-C30 Questionnaire. [file 13063_2019_3972_MOESM3_ESM.pdf]

EORTC QLQ-C30 (version 3)

我們有興趣知道一些您和您的健康的事情。請親自地回答所有問題，並圈出最切合您的情況的一個數字。答案沒有對與錯之分。您提供的所有資料將絕對保密。

請填寫您的縮寫名: |_|_|_|_|

您的出生日期 (日、月、年): |_|_|_|_|_|_|_|_|

今天日期 (日、月、年): 31 |_|_|_|_|_|_|_|_|

		完全沒有	有一點	頗多	非常多
1.	您做劇烈活動時會感到困難嗎，例如要提著很重的購物袋或手提箱？	1	2	3	4
2.	作遠距離的步行您會感到困難嗎？	1	2	3	4
3.	在屋外作近距離的步行您會感到困難嗎？	1	2	3	4
4.	您是否需要在白天時躺在床上或坐在椅子上？	1	2	3	4
5.	您是否需要別人協助吃飯、穿衣、洗澡或如廁？	1	2	3	4

**在過去一個星期內：**		**完全沒有**	**有一點**	**頗多**	**非常多**
6.	您做工作或其他日常活動時是否受到限制？	1	2	3	4
7.	您在追求自己的嗜好或其他休閒活動時是否受到限制？	1	2	3	4
8.	您是否感到氣促？	1	2	3	4
9.	您曾感到疼痛嗎？	1	2	3	4
10.	您需要休息嗎？	1	2	3	4
11.	您曾感到難以入睡嗎？	1	2	3	4
12.	您有感到軟弱無力嗎？	1	2	3	4
13.	您有感到沒胃口嗎？	1	2	3	4
14.	您有感到噁心嗎？	1	2	3	4
15.	您曾嘔吐嗎？	1	2	3	4
16.	您曾便秘嗎？	1	2	3	4

請轉下頁繼續

在過去一個星期內	完全沒有	有一點	頗多	非常多
17. 您曾腹瀉嗎？	1	2	3	4
18. 您是否感到疲倦？	1	2	3	4
19. 您的疼痛有干擾您的日常活動嗎？	1	2	3	4
20. 您曾覺得無法集中精神嗎，例如在讀報或看電視時？	1	2	3	4
21. 您感到緊張嗎？	1	2	3	4
22. 您感到擔憂嗎？	1	2	3	4
23. 您感到煩躁嗎？	1	2	3	4
24. 您感到沮喪嗎？	1	2	3	4
25. 對於記憶事情，您曾感到困難嗎？	1	2	3	4
26. 您的身體狀況或藥物治療有干擾您的家庭生活嗎？	1	2	3	4
27. 您的身體狀況或藥物治療有干擾您的社交活動嗎？	1	2	3	4
28. 您的身體狀況或藥物治療有否使到您的經濟出現困難？	1	2	3	4

在以下問題，請從 1 至 7 之間的數字圈選最切合您情況的答案。

29. 您對過去一星期內您的整體健康如何評分？

1	2	3	4	5	6	7
十分差						十分好

30. 您對過去一星期內您的整體生活質素如何評分？

1	2	3	4	5	6	7
十分差						十分好

© Copyright 1995 EORTC Quality of Life Group. All rights reserved. Version 3.0
