# Supplementary material for: The efficacy and safety of electro-acupuncture for alleviating chemotherapy-induced peripheral neuropathy in patients with coloreactal cancer: study protocol for a single-blinded, randomized sham-controlled trial
Source: Trials. 2020 Jan 9;21:58. doi: 10.1186/s13063-019-3972-5 (PMC6953283; doi:10.1186/s13063-019-3972-5)
Supplement: Supplementary file 4 — Additional file 4: CCMQ Questionnaire. [file 13063_2019_3972_MOESM4_ESM.pdf]

CCMQ 中醫體質量表（香港版）

本問卷是為了調查與你的體質有關的一些情況，從而為今後你的健康管理和臨床診治等提供參考。請閱讀每條問題，根據自己最近三個月內的實際情況或感覺，圈出最符合你的答案。如果你未能肯定如何回答某條問題時，請選擇最接近你實際情況的答案。

請注意：所有問題都是根據你最近三個月內的情況作答，而且每條問題只能選擇一個答案。

香港版之版權及使用權由香港醫院管理局持有，未經許可，不得擅用。

請根據最近三個月內的身體狀況和感覺，回答以下問題。	沒有<br>(或不會)	很少	有時	大部份<br>時間	常常
(1) 你精力充沛嗎？	1	2	3	4	5
(2) 你容易疲倦嗎？	1	2	3	4	5
(3) 你容易感到不够氣（ 呼吸急促，上氣不接下氣) 嗎？	1	2	3	4	5
(4) 你容易心慌嗎？	1	2	3	4	5
(5) 你容易頭暈或站起時暈眩嗎？	1	2	3	4	5
(6) 你喜歡安靜、懶得說話嗎?	1	2	3	4	5
(7) 你說話的聲音虛弱無力嗎?	1	2	3	4	5
(8) 你感到悶悶不樂、情緒低落嗎?	1	2	3	4	5

請根據最近三個月內的體驗和感覺，回答以下問題。	**沒有<br>(或不會)**	**很少**	**有時**	**經常**	**常常**

(9) 你容易精神緊張、焦慮不安嗎?	1	2	3	4	5
(10) 你多愁善感、容易感到悲傷嗎？	1	2	3	4	5
(11) 你容易感到害怕或受驚嗎?	1	2	3	4	5
(12) 你肋骨部位或乳房脹痛嗎?	1	2	3	4	5
(13) 你感到胸悶或腹部脹滿嗎？	1	2	3	4	5
(14) 你會無緣無故歎氣嗎?	1	2	3	4	5
(15) 你感到身體沉重不輕鬆嗎?	1	2	3	4	5
(16) 你感到手心或腳掌心發熱嗎?	1	2	3	4	5
(17) 你手腳凍嗎?	1	2	3	4	5
(18) 你胃部、背部或腰膝部位怕冷嗎？	1	2	3	4	5
(19) 你怕冷、衣服**較其他人**穿得多嗎?	1	2	3	4	5
(20) **你沒有生病的情況下,** 你會感到身體、臉上發熱嗎？	1	2	3	4	5
(21) 你**比一般人**較不能抵受寒冷(如冬天的寒冷，夏天的空調冷氣、電扇等)嗎？	1	2	3	4	5
(22) 你**比其他人**較容易感冒嗎?	1	2	3	4	5

請根據最近三個月內的體驗和感覺，回答以下問題。	**沒有 (或不會)**	**很少**	**有時**	**經常**	**常常**
(23) 你**沒有感冒時**也會打噴嚏嗎？	1	2	3	4	5

(24) 你**沒有感冒時**也會鼻塞、流鼻涕嗎?	1	2	3	4	5
(25) 你有**因季節變化、溫度變化或異味等原因**而咳嗽或喘氣的現象嗎？	1	2	3	4	5
(26) 你**活動量稍大**就容易出**虛汗**嗎?<br>(虛汗的意思是指比一般人容易大量出汗)	1	2	3	4	5
(27) 你容易忘記事情（健忘）嗎？	1	2	3	4	5
(28) 你有額頭油脂分泌多的現象嗎?	1	2	3	4	5
(29) 你口唇的顏色**比一般人**較紅嗎?	1	2	3	4	5
(30) 你容易過敏(對藥物、食物、氣味、花粉或在季節交替、氣候變化時)嗎?	1	2	3	4	5
(31) 你的皮膚容易起風疹(包括風團、風疹塊、風疙瘩)嗎?	1	2	3	4	5
(32) 你的皮膚**因過敏**出現紫红斑(即紫紅色瘀點、瘀斑)嗎?	1	2	3	4	5
(33) 你的皮膚**在不知不覺中**會出現青紫瘀斑(皮下出血)嗎?	1	2	3	4	5
(34) 你的皮膚一抓就紅，並出現抓痕嗎?	1	2	3	4	5
(35) 你皮膚或口唇乾嗎?	1	2	3	4	5
(36) 你面上顴骨兩面有細微紅絲嗎?	1	2	3	4	5

請根據最近三個月內的體驗和感覺，回答以下問題。	**沒有<br>(或不會)**	**很少**	**有時**	**經常**	**常常**
(37) 你身體上有哪裡疼痛嗎？	1	2	3	4	5
(38) 你面部**兩顴**泛紅或偏紅嗎?	1	2	3	4	5
(39) 你面部或鼻部有油膩感或者油亮發光嗎?	1	2	3	4	5

(40) 你面色暗淡、或容易出現灰褐色斑嗎?	1	2	3	4	5
(41) 你易生粉刺、暗瘡或膿瘡嗎?	1	2	3	4	5
(42) 你上眼瞼**比別人**較腫（上眼瞼有輕微隆起的現象）嗎?	1	2	3	4	5
(43) 你容易有黑眼圈嗎?	1	2	3	4	5
(44) 你感到眼睛乾澀嗎？	1	2	3	4	5
(45) 你口唇顏色偏暗嗎?	1	2	3	4	5
(46) 你感到口乾喉燥、總想喝水嗎？	1	2	3	4	5
(47) 你覺得喉嚨部位有異物感，而且吐不出來，也無法吞下嗎？	1	2	3	4	5
(48) 你感到口苦或口腔有異味嗎?	1	2	3	4	5
(49) 你口腔有黏黏的感覺嗎?	1	2	3	4	5
(50) 你腹部肥滿鬆軟嗎?	1	2	3	4	5

請根據最近三個月內的體驗和感覺，回答以下問題。	**沒有 (或不會)**	**很少**	**有時**	**經常**	**常常**
(51) 你平時痰多，特別是喉嚨部位總感到有痰堵塞著嗎?	1	2	3	4	5
(52) 你**吃(喝)涼的食物**時會感到不舒服或者怕吃(喝)涼的食物嗎？	1	2	3	4	5
(53) 你能適應自然和社會環境的外在變化嗎？	1	2	3	4	5
(54) 你容易失眠嗎？	1	2	3	4	5

(55) 你<u>**著涼或吃(喝)涼的食物後**</u>，容易腹瀉嗎?	1	2	3	4	5
(56) 你的大便黏糊不爽、有未排清的感覺嗎?	1	2	3	4	5
(57) 你容易便秘或大便乾燥嗎?	1	2	3	4	5
(58) 你舌苔厚膩或有舌苔厚厚的感覺嗎?	1	2	3	4	5
(59) 你小便時尿道發熱、尿色深黃嗎？	1	2	3	4	5
(60) 你的「白帶」顏色偏黃嗎?（**限女性回答**）	1	2	3	4	5
(60) 你的陰囊部位潮濕嗎? （**限男性回答**）	1	2	3	4	5
