# Supplementary material for: The efficacy and safety of electro-acupuncture for alleviating chemotherapy-induced peripheral neuropathy in patients with coloreactal cancer: study protocol for a single-blinded, randomized sham-controlled trial
Source: Trials. 2020 Jan 9;21:58. doi: 10.1186/s13063-019-3972-5 (PMC6953283; doi:10.1186/s13063-019-3972-5)
Supplement: Supplementary file 5 — Additional file 5: Light Touch Test and Vibration Test. [file 13063_2019_3972_MOESM5_ESM.pdf]

Light touch test and vibration test

Light touch test

Right hand<br>1. Yes / No<br>2. Yes / No<br>3. Yes / No	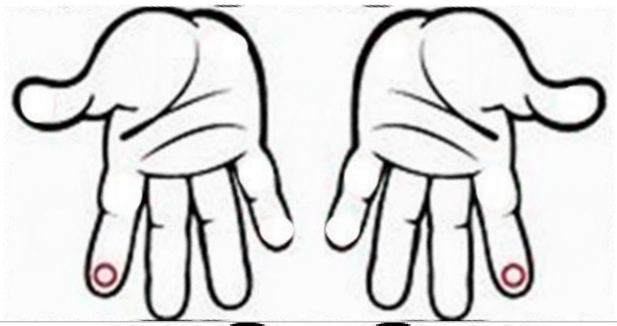	Left hand<br>1. Yes / No<br>2. Yes / No<br>3. Yes / No
Right foot<br>1. Yes / No<br>2. Yes / No<br>3. Yes / No	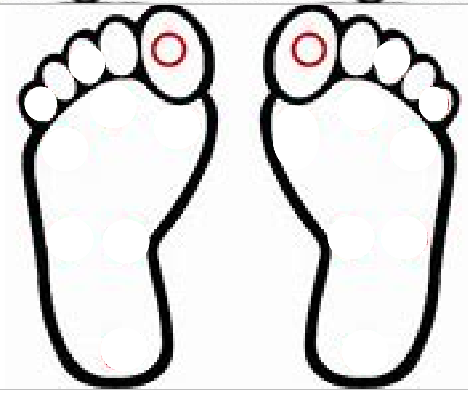	Left foot<br>1. Yes / No<br>2. Yes / No<br>3. Yes / No

Study number: ______________

Signed by recorder: ________________

Date: ______________________

Vibration test

Right hand<br>DIP joint<br>1. Yes / No<br>Reading: ______<br>2. Yes / No<br>Reading: ______<br>3. Yes / No<br>Reading: ______	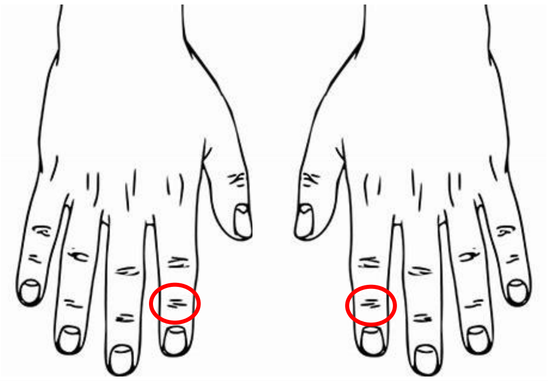	Left hand<br>DIP joint<br>1. Yes / No<br>Reading: ______<br>2. Yes / No<br>Reading: ______<br>3. Yes / No<br>Reading: ______
Right foot<br>IP of hallux<br>1. Yes / No<br>Reading: ______<br>2. Yes / No<br>Reading: ______<br>3. Yes / No<br>Reading: ______	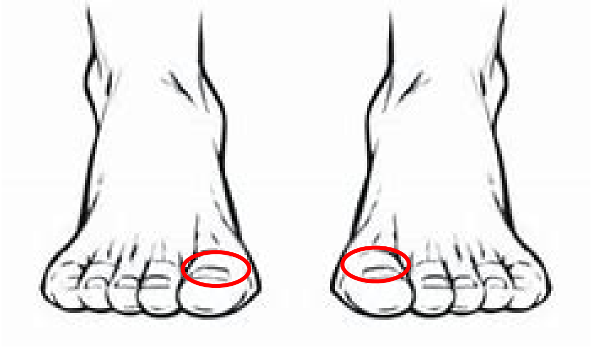	Left foot<br>IP of hallux<br>1. Yes / No<br>Reading: ______<br>2. Yes / No<br>Reading: ______<br>3. Yes / No<br>Reading: ______

Study number: _________________

Signed by recorder: _________________

Date: ________________________
