# Supplementary material for: The efficacy and safety of electro-acupuncture for alleviating chemotherapy-induced peripheral neuropathy in patients with coloreactal cancer: study protocol for a single-blinded, randomized sham-controlled trial
Source: Trials. 2020 Jan 9;21:58. doi: 10.1186/s13063-019-3972-5 (PMC6953283; doi:10.1186/s13063-019-3972-5)
Supplement: Supplementary file 6 — Additional file 6: Information Sheet and Consent Form. [file 13063_2019_3972_MOESM6_ESM.pdf]

仁濟醫院暨香港浸會大學中醫診所及臨床教研中心(下葵涌)、
瑪嘉烈醫院腫瘤科及
浸會大學香港中醫藥臨床研究中心

研究參加者資訊函

研究題目:

針灸對於預防化療引起的周邊神經病變的療效: 隨機對照試驗

研究參加者資訊函版本: VER 3.0 09/04/2018
研究編號:
參加者代號:

我們邀請您參與一項有關針灸預防化療引起的周邊神經病變的研究計劃。周邊神經病變的症狀包括手腳麻痺刺痛、功能下降(如扣鈕扣有困難)、感覺減弱(難以感覺手中小物體的形狀)等。在您決定參加之前，請您清楚明白這項研究的目的及其包括事項。請花時間仔細閱讀以下資料。如有需要，也請您與您的朋友，親人或家庭醫生討論。如有任何疑問，或想知道更多的資訊，請向我們查詢。

研究目的

這項研究目的是要評估針灸對於預防化療引起的周邊神經病變的臨床療效。

測試項目

是次研究會測試針灸的療效。針灸的運用已有長久歷史，普遍中醫師都以針灸治療不同的疾病，包括治療手腳麻痺疼痛。

參加者

診斷腸癌，並計劃接受化療，化療藥物必須包含奧沙利鉑(Oxaliplatin)的患者會被邀請參與此研究。

參與研究

您可以自由決定是否參與這項研究。如果您決定參與，我們將給予您這份資訊函作為保存並請簽署一份同意書。另外，如果您決定參與，您仍可以在任何時候無需給予任何理由退出，您的退出將不會影響您所接受的服務，亦不會影響您日後和仁濟醫院暨香港浸會大學中醫診所及臨床教研中心(下葵涌)及瑪嘉烈醫院腫瘤科的關係，以及將不影響您應得的醫療及法律權益。

研究方法

這是一個隨機單盲對照試驗，我們目標招募 84 人。實驗會分成兩組，針灸及假針灸的比率為 1:1。

參加者會被編成不同組別去比較。您會以抽籤方式編配至兩組的其中一組，被分配至針灸治療組的機會二分之一，而被分配至假針灸治療組的機會亦是二分之一。

仁濟醫院暨香港浸會大學中醫診所及臨床教研中心(下葵涌)、
瑪嘉烈醫院腫瘤科及
浸會大學香港中醫藥臨床研究中心

研究程式

此項研究為期 24 週。如果您決定參加這個研究，您需要先進行是次的篩選，接受 1 次完整的評估，然後接受為期十二週的針灸治療，再進行 3週1 次約 30 分鐘的治療後電話評估。

週數	0	1至12	12至24
時期	治療前	治療	治療後
覆診	接受一次篩選	一週一次針灸	評估

在第一次到診時，您要填寫 1 份問卷。您會被詳細詢問您的醫療記錄及最近的藥物治療。在第一次篩選時，我們會替您做一個身體檢查以記錄您的高度、體重和心率。此外，個人資料例如出生日期、性別、教育程度、婚姻狀況、年收入、就業情況都會在第一次篩選時記錄。

您將會接受每週兩次，每次約二十五分鐘，為期十二週的針灸治療。於完成治療後的十二星期，每三週電話或面談訪問您的身體情況，以評估您的周邊神經病變狀況。

日常生活限制

於研究期間(12 週)，您會被要求停止自行服食所有可能會治療周邊神經病變的中草藥、中成藥及相關針灸治療。與此同時，若西醫或中醫向您處方可能會治療周邊神經病變的藥物，例如維他命B、利痛抑(Lyrica / Pregabalin)、善痛眠(Neurontin / Gabapentin)或中藥 等，請您通知研究員以作記錄，我們會根據您的病情決定是否終止研究。若您有機會懷孕， 請於研究期間採取有效的避孕措施。若然懷孕，您將會從研究中排除。

治療費用及報酬

參加是次研究計劃時所接受的針灸治療是免費的。是次研究計劃不會提供任何型式的報酬。由於受研究經費的限制，研究完成後，假如您仍然希望繼續針灸治療，就需要按照本中心診療費用收費。

其他治療選擇

傳統治療周邊神經病變的藥物，例如維他命B、利痛抑(Lyrica / Pregabalin)、善痛眠(Neurontin / Gabapentin)等一般都可以經西醫處方或自行買到。

參加的副作用

所有醫療方式都可能會有副作用。

有些病人在接受治療時會有頭暈、疲勞、皮下血腫或局部感染。但所有不適都是暫時性

及不會引起嚴重後遺症。如果您在針灸後感到不適，你應該通知研究員。
此外，我們也會向您或您的醫生查詢每次化療前的常規血液檢查(血常規、肝功能、腎功能等)，以確保您沒有不良反應。

終止研究

假如您1)未能配合隨機分組安排，2)在研究中無故失訪，3)基於您的益處、安全或療效考慮，例如癌症病情惡化， 4)懷孕，以及5) 出現嚴重不良反應，您將會從研究中排除，終止您繼續參與這次研究。

參加的風險及不好處

針灸對孕婦未必安全，所以孕婦不能參與是次研究。分娩年齡及性生活活躍的女士，需要在研究期間採用有效的避孕措施。若果在研究期間發現自己懷孕，應該立即通知研究員。

參加的好處

針灸可能能預防您的周邊神經病變狀況及改善您的生活素質，但是這是不能保證的。是次計劃所收集的資料能夠增加我們對針灸治療化療引起周邊神經病變的認識。

新發現

在研究期間，若有新的發現會影響您繼續參加本研究的意願，研究員將會盡快告訴您。

若出了問題

針灸是安全的治療而且在中國有超過二千年的歷史。在香港，針灸亦廣為中醫師採用以治療不同的疾病。是次研究所選擇的針灸穴位都是中醫師用以治療手腳麻痺疼痛，故都是安全的。因參加本研究接受針灸治療而導致有嚴重身體創傷是不太可能會發生。若您真的發生任何事故，請緊記立即通知研究員，研究員會按照《醫院管理局針灸操作安全指引》進行相關處理。研究員會視乎情況而決定是否讓您繼續參加此研究。

試驗結束的規定

每個研究參與者都會得知研究的一般結果，如果您發現試驗治療能改善您的周邊神經病變狀況，您可以通知研究員，我們會在整個試驗結束後轉介您到一些能提供試驗治療的中心，但我們只會在您完成整個試驗後透露您在試驗中獲得甚麼。

資料保密

有需要的話，每個研究參與者都有權利獲得其個人的數據以及公開報告的研究結果。
根據香港法律（特別是「個人資料（私隱）條例」，第486 章），您有權對您個人資料進行保密，如在本項研究中或與本項研究有關的個人資料的收集、保管、保留、管理、控制、使用（分析或比較）、在香港內外轉讓、不披露、消除和/或任何方式處理。如有任何問題，您可以諮詢隱私資料私隱專員或致電到其辦公室（電話號碼：2827 2827），以適當監管或監督您個人資料保護，以便您能完全認識和瞭解確保遵守法律保護隱私資料的意義。
同意參與該項研究，您明確作出以下授權:

- 為了監督該項研究，授權主要研究者及其研究團隊和倫理委員根據本項研究和本知情同意書規定的方式獲得、使用並保留您的個人資料，並且
- 為了檢查和核實研究資料的完整性、評估研究協定與相關要求的一致性，授權相關的政府機構（如香港衛生署）可獲得您個人資料。

研究期間所有收集到關於您的資料都會絕對保密。您的資料只會用於是次研究。研究所得的資料會存放三年，之後便會銷毀。

仁濟醫院暨香港浸會大學中醫診所及臨床教研中心(下葵涌)、
瑪嘉烈醫院腫瘤科及
浸會大學香港中醫藥臨床研究中心

主要研究員

是次研究由仁濟醫院暨香港浸會大學中醫診所及臨床教研中心(下葵涌)陳啟賢中醫師、瑪嘉烈醫院腫瘤科呂卓如醫生及浸會大學香港中醫藥臨床研究中心鍾麗丹博士安排。

誰覆閱過這項研究？

醫管局九龍西醫院聯網研究倫理委員會曾審閱及批准此研究。

有關更多資訊的聯絡

如您在研究期間有突發事件，或感到不適，請致電我們研究小組的熱線：2370 2216。如您需要更多研究有關資訊或有疑問，請聯絡陳啟賢中醫師，電話 2370 2216。

若閣下參與時認為過程安排與上述不符或閣下參與的權利被觸犯時，您可以致電 2990 1017 聯絡「醫管局九龍西醫院聯網研究倫理委員會」。

多謝您參與這項研究，這份研究資料的複本和您簽署的同意書將會給您保存。

仁濟醫院暨香港浸會大學中醫診所及臨床教研中心(下葵涌)、瑪嘉烈醫院腫瘤科及浸會大學香港中醫藥臨床研究中心

針灸須知

治療前

一・若有以下情況請告知醫師

1. 初次治療精神緊張或曾經有過暈針史者；
2. 女性正值月經、懷孕期；
3. 安裝心臟起搏器；
4. 正在服用以下藥物：薄血藥華法林（Warfarin）、阿司匹林等
5. 有以下病史

1. 嚴重心腦血管疾病：如：心肌梗塞、動脈瘤、中風等	5. 骨質疏鬆症；
2. 癲癇	6. 骨折（並有金屬支架固定）
3. 傳染病（乙型肝炎、愛滋病等）	7. 糖尿病控制不佳
4. 腫瘤患者	8. 各類傷口未癒合者

二・穿著寬鬆，以便需要時暴露身體穴位部分。

三・請勿空腹或飽餐。飢餓者可先進食少量食物。

治療中

一・治療全過程請關閉手提電話（必要時調至震動），注意治療反應，保持清醒，切勿睡著。

二・選取舒適體位，留針過程中請勿移動身體或改變姿勢，以免發生彎針或滯針。

三・照燈（TDP）治療時，局部皮膚感覺灼熱，請即時告知醫師。

四・有任何不適，如治療部位疼痛灼熱或胸悶欲嘔、眩暈、大量冒汗等，如無醫師在身邊，請即時按響平安鐘。

治療後

一・ 針刺處仍有刺痛感或症狀加重，請告知醫師，並稍坐片刻才離開診所。建議針灸後兩小時方可洗澡，治療當日避免冷水洗澡及游泳。

二・ 針刺處如有重脹或痠麻，是治療遺留針感的常見現象，可自行揉按會逐漸消失；拔針後，針刺部位有時出現青紫、腫脹或硬結，可按壓局部片刻；如青紫腫塊較明顯，可先作冷敷，24 小時後再熱敷，一般 3～5 天後慢慢消退。

三・ 照燈（TDP）治療後如有水泡，請盡快通知醫師處理。如離開本診所後才起水泡，請致電 2370 2216 通知醫師，並於本診所辦公時間找醫師求助。

仁濟醫院暨香港浸會大學中醫診所及臨床教研中心(下葵涌)、瑪嘉烈醫院腫瘤科及浸會大學香港中醫藥臨床研究中心

<u>知情同意書</u>

研究編號:
參加者代號:

研究題目：針灸對於預防化療引起周邊神經病變的療效: 隨機對照試驗

研究員姓名：陳啟賢中醫師、呂卓如醫生及鍾麗丹博士

1. 我確定我已經閱讀及明白研究參加者資訊函，並有機會提出任何問題。

2. 我已被知會及明白此臨床研究的性質、我的責任及自願參與此研究可能帶來的不好處。我已收到所有有關是次研究的書面資料(研究參加者資訊函乙份、針灸須知乙份)。

3. 我明白我可自由參與並且可在任何時候自由退出，這將不影響我應得的醫療服務及法律權益。

4. 我明白研究人員和管理研究當局會翻閱我有關參與研究的醫療紀錄。我同意授權有關人員翻查我的醫療紀錄。

5. 我同意參與上述研究。

6. 我明白我會收到一份已簽名及有日期的知情同意書。

參加者姓名	日期	簽署
見證人姓名 (如適用)	日期	簽署
研究員姓名	日期	簽署

複本呈：參加者
研究員檔案
